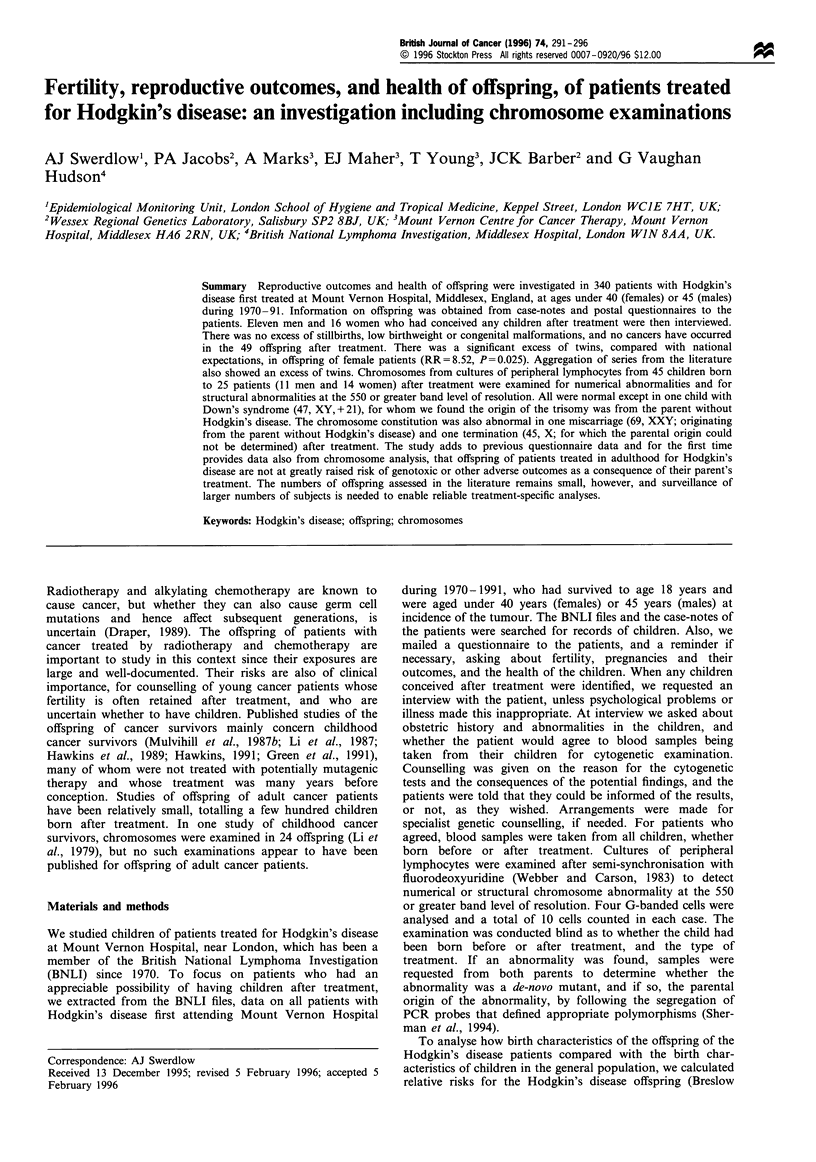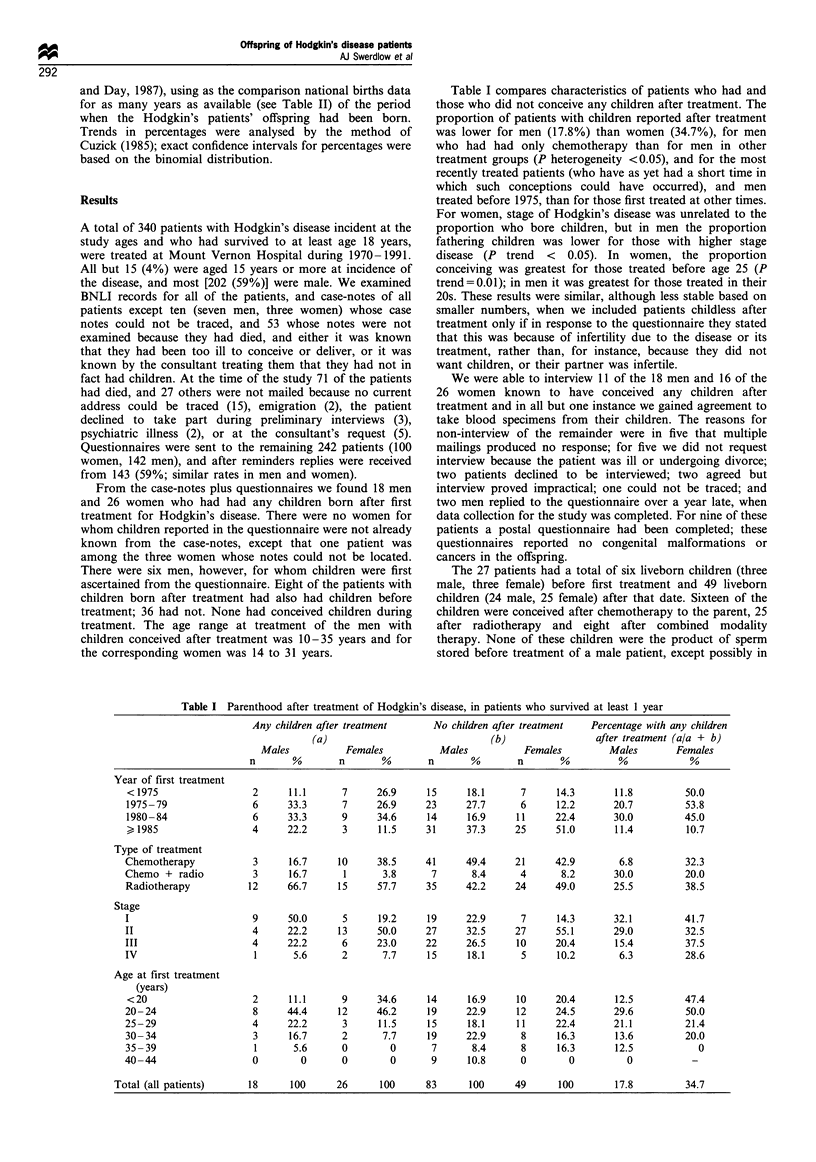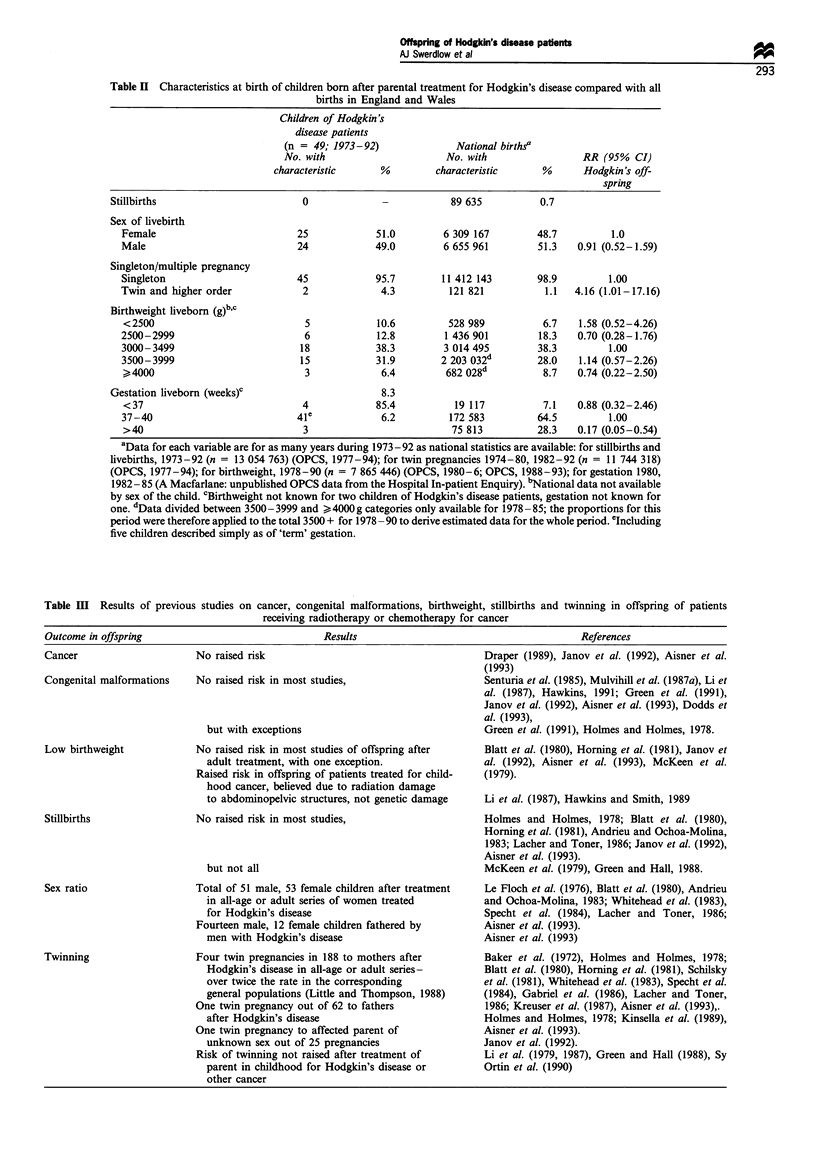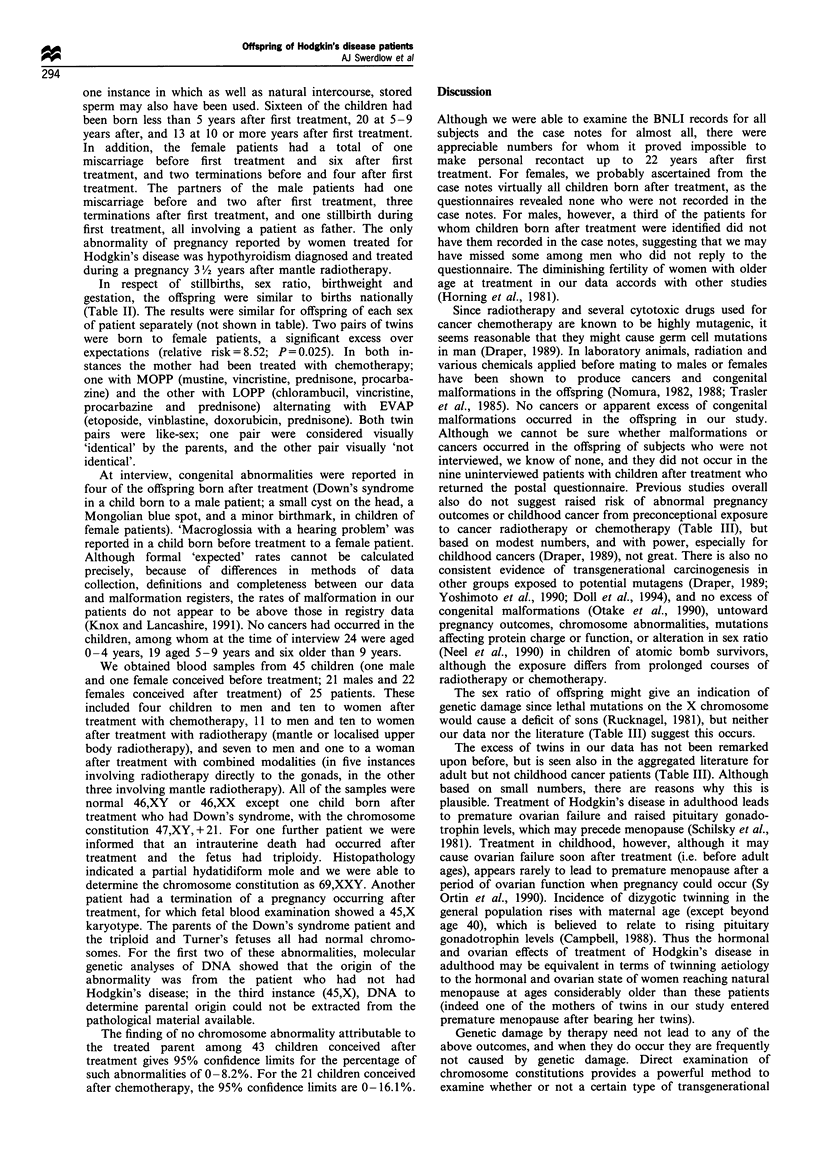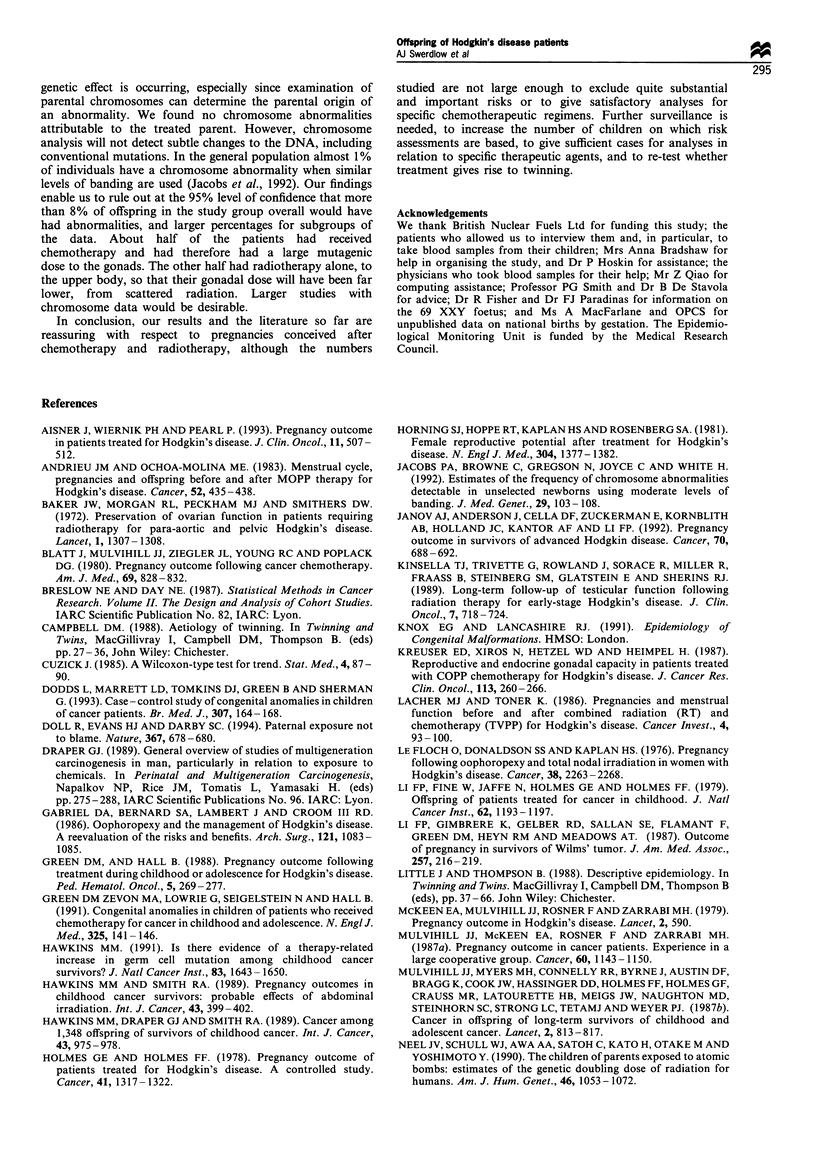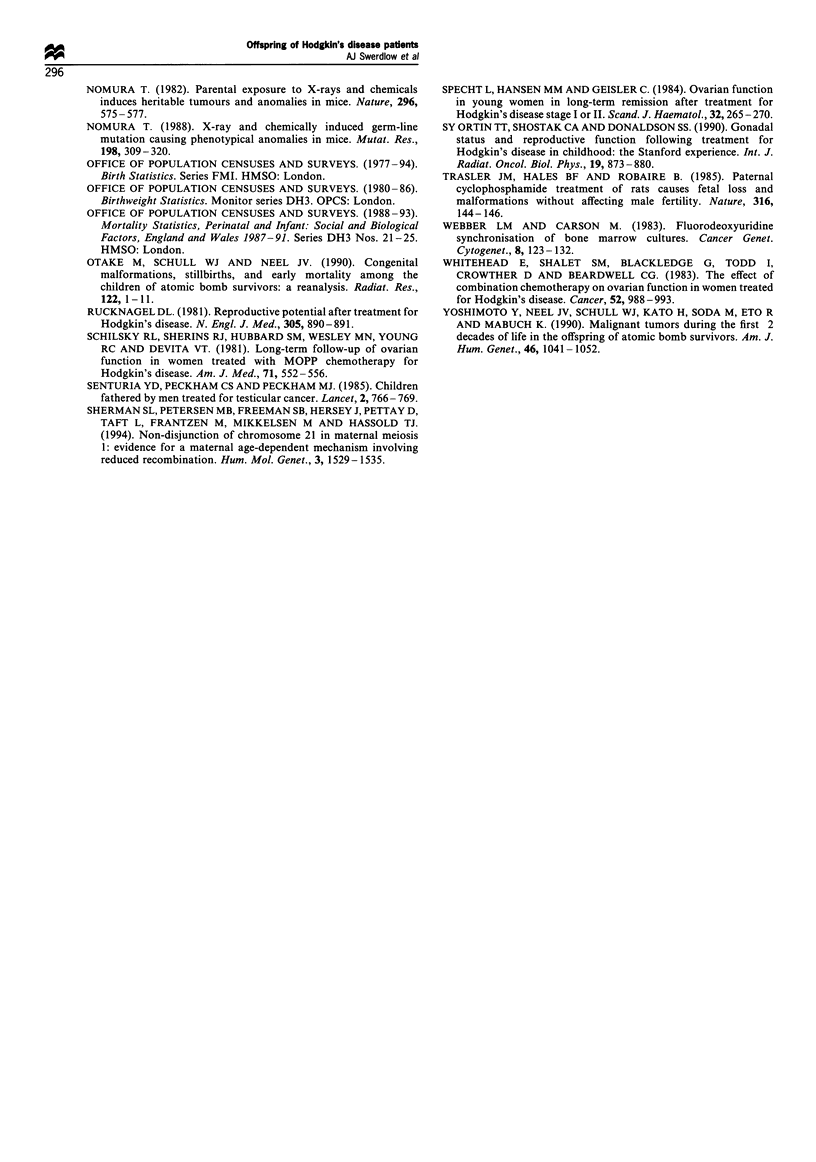# Fertility, reproductive outcomes, and health of offspring, of patients treated for Hodgkin's disease: an investigation including chromosome examinations.

**DOI:** 10.1038/bjc.1996.355

**Published:** 1996-07

**Authors:** A. J. Swerdlow, P. A. Jacobs, A. Marks, E. J. Maher, T. Young, J. C. Barber, G. Vaughan Hudson

**Affiliations:** Epidemiological Monitoring Unit, London School of Hygiene and Tropical Medicine, UK.

## Abstract

Reproductive outcomes and health of offspring were investigated in 340 patients with Hodgkin's disease first treated at Mount Vernon Hospital, Middlesex, England, at ages under 40 (females) or 45 (males) during 1970-91. Information on offspring was obtained from case-notes and postal questionnaires to the patients. Eleven men and 16 women who had conceived any children after treatment were then interviewed. There was no excess of stillbirths, low birthweight or cogenital malformations, and no cancers have occurred in the 49 offspring after treatment. There was a significant excess of twins, compared with national expectations, in offspring of female patients (RR = 8.52, P = 0.025). Aggregation of series from the literature also showed an excess of twins. Chromosomes from cultures of peripheral lymphocytes from 45 children born to 25 patients (11 men and 14 women) after treatment were examined for numerical abnormalities and for structural abnormalities at the 550 or greater band level of resolution. All were normal except in one child with Down's syndrome (47, XY, +21), for whom we found the origin of the trisomy was from the parent without Hodgkin's disease. The chromosome constitution was also abnormal in one miscarriage (69, XXY; originating from the parent without Hodgkin's disease) and one termination (45, X; for with the parental origin could not be determined) after treatment. The study adds to previous questionnaire data and for the first time provides data also from chromosome analysis, that offspring of patients treated in adulthood for Hodgkin's disease are not at greatly raised risk of genotoxic or other adverse outcomes as a consequence of their parent's treatment. The numbers of offspring assessed in the literature remains small, however, and surveillance of larger numbers of subjects is needed to enable reliable treatment-specific analyses.


British Journal of Cancer (1996) 74, 291-296
© 1996 Stockton Press All rights reserved 0007-0920/96 $12.00

### Fertility, reproductive outcomes, and health of offspring, of patients treated for Hodgkin's disease: an investigation including chromosome examinations

AJ Swerdlow[1], PA Jacobs[2], A Marks[3], EJ Maher[3], T Young[3], JCK Barber[2] and G Vaughan Hudson[4]

[1]*Epidemiological Monitoring Unit, London School of Hygiene and Tropical Medicine, Keppel Street, London WC1E 7HT, UK;* [2]*Wessex Regional Genetics Laboratory, Salisbury SP2 8BJ, UK;* [3]*Mount Vernon Centre for Cancer Therapy, Mount Vernon Hospital, Middlesex HA6 2RN, UK;* [4]*British National Lymphoma Investigation, Middlesex Hospital, London W1N 8AA, UK.*

**Summary** Reproductive outcomes and health of offspring were investigated in 340 patients with Hodgkin's disease first treated at Mount Vernon Hospital, Middlesex, England, at ages under 40 (females) or 45 (males) during 1970–91. Information on offspring was obtained from case-notes and postal questionnaires to the patients. Eleven men and 16 women who had conceived any children after treatment were then interviewed. There was no excess of stillbirths, low birthweight or congenital malformations, and no cancers have occurred in the 49 offspring after treatment. There was a significant excess of twins, compared with national expectations, in offspring of female patients (RR = 8.52, $P = 0.025$). Aggregation of series from the literature also showed an excess of twins. Chromosomes from cultures of peripheral lymphocytes from 45 children born to 25 patients (11 men and 14 women) after treatment were examined for numerical abnormalities and for structural abnormalities at the 550 or greater band level of resolution. All were normal except in one child with Down's syndrome (47, XY, +21), for whom we found the origin of the trisomy was from the parent without Hodgkin's disease. The chromosome constitution was also abnormal in one miscarriage (69, XXY; originating from the parent without Hodgkin's disease) and one termination (45, X; for which the parental origin could not be determined) after treatment. The study adds to previous questionnaire data and for the first time provides data also from chromosome analysis, that offspring of patients treated in adulthood for Hodgkin's disease are not at greatly raised risk of genotoxic or other adverse outcomes as a consequence of their parent's treatment. The numbers of offspring assessed in the literature remains small, however, and surveillance of larger numbers of subjects is needed to enable reliable treatment-specific analyses.

**Keywords:** Hodgkin's disease; offspring; chromosomes

Radiotherapy and alkylating chemotherapy are known to cause cancer, but whether they can also cause germ cell mutations and hence affect subsequent generations, is uncertain (Draper, 1989). The offspring of patients with cancer treated by radiotherapy and chemotherapy are important to study in this context since their exposures are large and well-documented. Their risks are also of clinical importance, for counselling of young cancer patients whose fertility is often retained after treatment, and who are uncertain whether to have children. Published studies of the offspring of cancer survivors mainly concern childhood cancer survivors (Mulvihill *et al.*, 1987*b*; Li *et al.*, 1987; Hawkins *et al.*, 1989; Hawkins, 1991; Green *et al.*, 1991), many of whom were not treated with potentially mutagenic therapy and whose treatment was many years before conception. Studies of offspring of adult cancer patients have been relatively small, totalling a few hundred children born after treatment. In one study of childhood cancer survivors, chromosomes were examined in 24 offspring (Li *et al.*, 1979), but no such examinations appear to have been published for offspring of adult cancer patients.

### Materials and methods

We studied children of patients treated for Hodgkin's disease at Mount Vernon Hospital, near London, which has been a member of the British National Lymphoma Investigation (BNLI) since 1970. To focus on patients who had an appreciable possibility of having children after treatment, we extracted from the BNLI files, data on all patients with Hodgkin's disease first attending Mount Vernon Hospital during 1970–1991, who had survived to age 18 years and were aged under 40 years (females) or 45 years (males) at incidence of the tumour. The BNLI files and the case-notes of the patients were searched for records of children. Also, we mailed a questionnaire to the patients, and a reminder if necessary, asking about fertility, pregnancies and their outcomes, and the health of the children. When any children conceived after treatment were identified, we requested an interview with the patient, unless psychological problems or illness made this inappropriate. At interview we asked about obstetric history and abnormalities in the children, and whether the patient would agree to blood samples being taken from their children for cytogenetic examination. Counselling was given on the reason for the cytogenetic tests and the consequences of the potential findings, and the patients were told that they could be informed of the results, or not, as they wished. Arrangements were made for specialist genetic counselling, if needed. For patients who agreed, blood samples were taken from all children, whether born before or after treatment. Cultures of peripheral lymphocytes were examined after semi-synchronisation with fluorodeoxyuridine (Webber and Carson, 1983) to detect numerical or structural chromosome abnormality at the 550 or greater band level of resolution. Four G-banded cells were analysed and a total of 10 cells counted in each case. The examination was conducted blind as to whether the child had been born before or after treatment, and the type of treatment. If an abnormality was found, samples were requested from both parents to determine whether the abnormality was a *de-novo* mutant, and if so, the parental origin of the abnormality, by following the segregation of PCR probes that defined appropriate polymorphisms (Sherman *et al.*, 1994).

To analyse how birth characteristics of the offspring of the Hodgkin's disease patients compared with the birth characteristics of children in the general population, we calculated relative risks for the Hodgkin's disease offspring (Breslow

Correspondence: AJ Swerdlow
Received 13 December 1995; revised 5 February 1996; accepted 5 February 1996

and Day, 1987), using as the comparison national births data for as many years as available (see Table II) of the period when the Hodgkin's patients' offspring had been born. Trends in percentages were analysed by the method of Cuzick (1985); exact confidence intervals for percentages were based on the binomial distribution.

### Results

A total of 340 patients with Hodgkin's disease incident at the study ages and who had survived to at least age 18 years, were treated at Mount Vernon Hospital during 1970–1991. All but 15 (4%) were aged 15 years or more at incidence of the disease, and most [202 (59%)] were male. We examined BNLI records for all of the patients, and case-notes of all patients except ten (seven men, three women) whose case notes could not be traced, and 53 whose notes were not examined because they had died, and either it was known that they had been too ill to conceive or deliver, or it was known by the consultant treating them that they had not in fact had children. At the time of the study 71 of the patients had died, and 27 others were not mailed because no current address could be traced (15), emigration (2), the patient declined to take part during preliminary interviews (3), psychiatric illness (2), or at the consultant's request (5). Questionnaires were sent to the remaining 242 patients (100 women, 142 men), and after reminders replies were received from 143 (59%; similar rates in men and women).

From the case-notes plus questionnaires we found 18 men and 26 women who had had any children born after first treatment for Hodgkin's disease. There were no women for whom children reported in the questionnaire were not already known from the case-notes, except that one patient was among the three women whose notes could not be located. There were six men, however, for whom children were first ascertained from the questionnaire. Eight of the patients with children born after treatment had also had children before treatment; 36 had not. None had conceived children during treatment. The age range at treatment of the men with children conceived after treatment was 10–35 years and for the corresponding women was 14 to 31 years.

Table I compares characteristics of patients who had and those who did not conceive any children after treatment. The proportion of patients with children reported after treatment was lower for men (17.8%) than women (34.7%), for men who had had only chemotherapy than for men in other treatment groups ($P$ heterogeneity $<0.05$), and for the most recently treated patients (who have as yet had a short time in which such conceptions could have occurred), and men treated before 1975, than for those first treated at other times. For women, stage of Hodgkin's disease was unrelated to the proportion who bore children, but in men the proportion fathering children was lower for those with higher stage disease ($P$ trend $<$ 0.05). In women, the proportion conceiving was greatest for those treated before age 25 ($P$ trend$=0.01$); in men it was greatest for those treated in their 20s. These results were similar, although less stable based on smaller numbers, when we included patients childless after treatment only if in response to the questionnaire they stated that this was because of infertility due to the disease or its treatment, rather than, for instance, because they did not want children, or their partner was infertile.

We were able to interview 11 of the 18 men and 16 of the 26 women known to have conceived any children after treatment and in all but one instance we gained agreement to take blood specimens from their children. The reasons for non-interview of the remainder were in five that multiple mailings produced no response; for five we did not request interview because the patient was ill or undergoing divorce; two patients declined to be interviewed; two agreed but interview proved impractical; one could not be traced; and two men replied to the questionnaire over a year late, when data collection for the study was completed. For nine of these patients a postal questionnaire had been completed; these questionnaires reported no congenital malformations or cancers in the offspring.

The 27 patients had a total of six liveborn children (three male, three female) before first treatment and 49 liveborn children (24 male, 25 female) after that date. Sixteen of the children were conceived after chemotherapy to the parent, 25 after radiotherapy and eight after combined modality therapy. None of these children were the product of sperm stored before treatment of a male patient, except possibly in

**Table I** Parenthood after treatment of Hodgkin's disease, in patients who survived at least 1 year

| | *Any children after treatment (a)* | | | | *No children after treatment (b)* | | | | *Percentage with any children after treatment (a/a + b)* | |
|---|---|---|---|---|---|---|---|---|---|---|
| | *Males* | | *Females* | | *Males* | | *Females* | | *Males* | *Females* |
| | n | % | n | % | n | % | n | % | % | % |
| Year of first treatment | | | | | | | | | | |
| <1975 | 2 | 11.1 | 7 | 26.9 | 15 | 18.1 | 7 | 14.3 | 11.8 | 50.0 |
| 1975–79 | 6 | 33.3 | 7 | 26.9 | 23 | 27.7 | 6 | 12.2 | 20.7 | 53.8 |
| 1980–84 | 6 | 33.3 | 9 | 34.6 | 14 | 16.9 | 11 | 22.4 | 30.0 | 45.0 |
| ⩾1985 | 4 | 22.2 | 3 | 11.5 | 31 | 37.3 | 25 | 51.0 | 11.4 | 10.7 |
| Type of treatment | | | | | | | | | | |
| Chemotherapy | 3 | 16.7 | 10 | 38.5 | 41 | 49.4 | 21 | 42.9 | 6.8 | 32.3 |
| Chemo + radio | 3 | 16.7 | 1 | 3.8 | 7 | 8.4 | 4 | 8.2 | 30.0 | 20.0 |
| Radiotherapy | 12 | 66.7 | 15 | 57.7 | 35 | 42.2 | 24 | 49.0 | 25.5 | 38.5 |
| Stage | | | | | | | | | | |
| I | 9 | 50.0 | 5 | 19.2 | 19 | 22.9 | 7 | 14.3 | 32.1 | 41.7 |
| II | 4 | 22.2 | 13 | 50.0 | 27 | 32.5 | 27 | 55.1 | 29.0 | 32.5 |
| III | 4 | 22.2 | 6 | 23.0 | 22 | 26.5 | 10 | 20.4 | 15.4 | 37.5 |
| IV | 1 | 5.6 | 2 | 7.7 | 15 | 18.1 | 5 | 10.2 | 6.3 | 28.6 |
| Age at first treatment (years) | | | | | | | | | | |
| <20 | 2 | 11.1 | 9 | 34.6 | 14 | 16.9 | 10 | 20.4 | 12.5 | 47.4 |
| 20–24 | 8 | 44.4 | 12 | 46.2 | 19 | 22.9 | 12 | 24.5 | 29.6 | 50.0 |
| 25–29 | 4 | 22.2 | 3 | 11.5 | 15 | 18.1 | 11 | 22.4 | 21.1 | 21.4 |
| 30–34 | 3 | 16.7 | 2 | 7.7 | 19 | 22.9 | 8 | 16.3 | 13.6 | 20.0 |
| 35–39 | 1 | 5.6 | 0 | 0 | 7 | 8.4 | 8 | 16.3 | 12.5 | 0 |
| 40–44 | 0 | 0 | 0 | 0 | 9 | 10.8 | 0 | 0 | 0 | – |
| Total (all patients) | 18 | 100 | 26 | 100 | 83 | 100 | 49 | 100 | 17.8 | 34.7 |

**Table II** Characteristics at birth of children born after parental treatment for Hodgkin's disease compared with all births in England and Wales

| | *Children of Hodgkin's disease patients (n = 49; 1973–92)* No. with characteristic | % | *National births*[a] No. with characteristic | % | *RR (95% CI) Hodgkin's off-spring* |
|---|---|---|---|---|---|
| Stillbirths | 0 | – | 89 635 | 0.7 | |
| Sex of livebirth | | | | | |
| Female | 25 | 51.0 | 6 309 167 | 48.7 | 1.0 |
| Male | 24 | 49.0 | 6 655 961 | 51.3 | 0.91 (0.52–1.59) |
| Singleton/multiple pregnancy | | | | | |
| Singleton | 45 | 95.7 | 11 412 143 | 98.9 | 1.00 |
| Twin and higher order | 2 | 4.3 | 121 821 | 1.1 | 4.16 (1.01–17.16) |
| Birthweight liveborn (g)[b,c] | | | | | |
| <2500 | 5 | 10.6 | 528 989 | 6.7 | 1.58 (0.52–4.26) |
| 2500–2999 | 6 | 12.8 | 1 436 901 | 18.3 | 0.70 (0.28–1.76) |
| 3000–3499 | 18 | 38.3 | 3 014 495 | 38.3 | 1.00 |
| 3500–3999 | 15 | 31.9 | 2 203 032[d] | 28.0 | 1.14 (0.57–2.26) |
| ⩾4000 | 3 | 6.4 | 682 028[d] | 8.7 | 0.74 (0.22–2.50) |
| Gestation liveborn (weeks)[c] | | 8.3 | | | |
| <37 | 4 | 85.4 | 19 117 | 7.1 | 0.88 (0.32–2.46) |
| 37–40 | 41[e] | 6.2 | 172 583 | 64.5 | 1.00 |
| >40 | 3 | | 75 813 | 28.3 | 0.17 (0.05–0.54) |

[a]Data for each variable are for as many years during 1973–92 as national statistics are available: for stillbirths and livebirths, 1973–92 ($n$ = 13 054 763) (OPCS, 1977–94); for twin pregnancies 1974–80, 1982–92 ($n$ = 11 744 318) (OPCS, 1977–94); for birthweight, 1978–90 ($n$ = 7 865 446) (OPCS, 1980–6; OPCS, 1988–93); for gestation 1980, 1982–85 (A Macfarlane: unpublished OPCS data from the Hospital In-patient Enquiry). [b]National data not available by sex of the child. [c]Birthweight not known for two children of Hodgkin's disease patients, gestation not known for one. [d]Data divided between 3500–3999 and ⩾4000 g categories only available for 1978–85; the proportions for this period were therefore applied to the total 3500+ for 1978–90 to derive estimated data for the whole period. [e]Including five children described simply as of 'term' gestation.

**Table III** Results of previous studies on cancer, congenital malformations, birthweight, stillbirths and twinning in offspring of patients receiving radiotherapy or chemotherapy for cancer

| *Outcome in offspring* | *Results* | *References* |
|---|---|---|
| Cancer | No raised risk | Draper (1989), Janov *et al.* (1992), Aisner *et al.* (1993) |
| Congenital malformations | No raised risk in most studies, | Senturia *et al.* (1985), Mulvihill *et al.* (1987*a*), Li *et al.* (1987), Hawkins, 1991; Green *et al.* (1991), Janov *et al.* (1992), Aisner *et al.* (1993), Dodds *et al.* (1993), |
| | but with exceptions | Green *et al.* (1991), Holmes and Holmes, 1978. |
| Low birthweight | No raised risk in most studies of offspring after adult treatment, with one exception. | Blatt *et al.* (1980), Horning *et al.* (1981), Janov *et al.* (1992), Aisner *et al.* (1993), McKeen *et al.* (1979). |
| | Raised risk in offspring of patients treated for child-hood cancer, believed due to radiation damage to abdominopelvic structures, not genetic damage | Li *et al.* (1987), Hawkins and Smith, 1989 |
| Stillbirths | No raised risk in most studies, | Holmes and Holmes, 1978; Blatt *et al.* (1980), Horning *et al.* (1981), Andrieu and Ochoa-Molina, 1983; Lacher and Toner, 1986; Janov *et al.* (1992), Aisner *et al.* (1993). |
| | but not all | McKeen *et al.* (1979), Green and Hall, 1988. |
| Sex ratio | Total of 51 male, 53 female children after treatment in all-age or adult series of women treated for Hodgkin's disease | Le Floch *et al.* (1976), Blatt *et al.* (1980), Andrieu and Ochoa-Molina, 1983; Whitehead *et al.* (1983), Specht *et al.* (1984), Lacher and Toner, 1986; Aisner *et al.* (1993). |
| | Fourteen male, 12 female children fathered by men with Hodgkin's disease | Aisner *et al.* (1993) |
| Twinning | Four twin pregnancies in 188 to mothers after Hodgkin's disease in all-age or adult series – over twice the rate in the corresponding general populations (Little and Thompson, 1988) | Baker *et al.* (1972), Holmes and Holmes, 1978; Blatt *et al.* (1980), Horning *et al.* (1981), Schilsky *et al.* (1981), Whitehead *et al.* (1983), Specht *et al.* (1984), Gabriel *et al.* (1986), Lacher and Toner, 1986; Kreuser *et al.* (1987), Aisner *et al.* (1993),. |
| | One twin pregnancy out of 62 to fathers after Hodgkin's disease | Holmes and Holmes, 1978; Kinsella *et al.* (1989), Aisner *et al.* (1993). |
| | One twin pregnancy to affected parent of unknown sex out of 25 pregnancies | Janov *et al.* (1992). |
| | Risk of twinning not raised after treatment of parent in childhood for Hodgkin's disease or other cancer | Li *et al.* (1979, 1987), Green and Hall (1988), Sy Ortin *et al.* (1990) |

one instance in which as well as natural intercourse, stored sperm may also have been used. Sixteen of the children had been born less than 5 years after first treatment, 20 at 5–9 years after, and 13 at 10 or more years after first treatment. In addition, the female patients had a total of one miscarriage before first treatment and six after first treatment, and two terminations before and four after first treatment. The partners of the male patients had one miscarriage before and two after first treatment, three terminations after first treatment, and one stillbirth during first treatment, all involving a patient as father. The only abnormality of pregnancy reported by women treated for Hodgkin's disease was hypothyroidism diagnosed and treated during a pregnancy 3½ years after mantle radiotherapy.

In respect of stillbirths, sex ratio, birthweight and gestation, the offspring were similar to births nationally (Table II). The results were similar for offspring of each sex of patient separately (not shown in table). Two pairs of twins were born to female patients, a significant excess over expectations (relative risk = 8.52; $P = 0.025$). In both instances the mother had been treated with chemotherapy; one with MOPP (mustine, vincristine, prednisone, procarbazine) and the other with LOPP (chlorambucil, vincristine, procarbazine and prednisone) alternating with EVAP (etoposide, vinblastine, doxorubicin, prednisone). Both twin pairs were like-sex; one pair were considered visually 'identical' by the parents, and the other pair visually 'not identical'.

At interview, congenital abnormalities were reported in four of the offspring born after treatment (Down's syndrome in a child born to a male patient; a small cyst on the head, a Mongolian blue spot, and a minor birthmark, in children of female patients). 'Macroglossia with a hearing problem' was reported in a child born before treatment to a female patient. Although formal 'expected' rates cannot be calculated precisely, because of differences in methods of data collection, definitions and completeness between our data and malformation registers, the rates of malformation in our patients do not appear to be above those in registry data (Knox and Lancashire, 1991). No cancers had occurred in the children, among whom at the time of interview 24 were aged 0–4 years, 19 aged 5–9 years and six older than 9 years.

We obtained blood samples from 45 children (one male and one female conceived before treatment; 21 males and 22 females conceived after treatment) of 25 patients. These included four children to men and ten to women after treatment with chemotherapy, 11 to men and ten to women after treatment with radiotherapy (mantle or localised upper body radiotherapy), and seven to men and one to a woman after treatment with combined modalities (in five instances involving radiotherapy directly to the gonads, in the other three involving mantle radiotherapy). All of the samples were normal 46,XY or 46,XX except one child born after treatment who had Down's syndrome, with the chromosome constitution 47,XY,+21. For one further patient we were informed that an intrauterine death had occurred after treatment and the fetus had triploidy. Histopathology indicated a partial hydatidiform mole and we were able to determine the chromosome constitution as 69,XXY. Another patient had a termination of a pregnancy occurring after treatment, for which fetal blood examination showed a 45,X karyotype. The parents of the Down's syndrome patient and the triploid and Turner's fetuses all had normal chromosomes. For the first two of these abnormalities, molecular genetic analyses of DNA showed that the origin of the abnormality was from the patient who had not had Hodgkin's disease; in the third instance (45,X), DNA to determine parental origin could not be extracted from the pathological material available.

The finding of no chromosome abnormality attributable to the treated parent among 43 children conceived after treatment gives 95% confidence limits for the percentage of such abnormalities of 0–8.2%. For the 21 children conceived after chemotherapy, the 95% confidence limits are 0–16.1%.

### Discussion

Although we were able to examine the BNLI records for all subjects and the case notes for almost all, there were appreciable numbers for whom it proved impossible to make personal recontact up to 22 years after first treatment. For females, we probably ascertained from the case notes virtually all children born after treatment, as the questionnaires revealed none who were not recorded in the case notes. For males, however, a third of the patients for whom children born after treatment were identified did not have them recorded in the case notes, suggesting that we may have missed some among men who did not reply to the questionnaire. The diminishing fertility of women with older age at treatment in our data accords with other studies (Horning *et al.*, 1981).

Since radiotherapy and several cytotoxic drugs used for cancer chemotherapy are known to be highly mutagenic, it seems reasonable that they might cause germ cell mutations in man (Draper, 1989). In laboratory animals, radiation and various chemicals applied before mating to males or females have been shown to produce cancers and congenital malformations in the offspring (Nomura, 1982, 1988; Trasler *et al.*, 1985). No cancers or apparent excess of congenital malformations occurred in the offspring in our study. Although we cannot be sure whether malformations or cancers occurred in the offspring of subjects who were not interviewed, we know of none, and they did not occur in the nine uninterviewed patients with children after treatment who returned the postal questionnaire. Previous studies overall also do not suggest raised risk of abnormal pregnancy outcomes or childhood cancer from preconceptional exposure to cancer radiotherapy or chemotherapy (Table III), but based on modest numbers, and with power, especially for childhood cancers (Draper, 1989), not great. There is also no consistent evidence of transgenerational carcinogenesis in other groups exposed to potential mutagens (Draper, 1989; Yoshimoto *et al.*, 1990; Doll *et al.*, 1994), and no excess of congenital malformations (Otake *et al.*, 1990), untoward pregnancy outcomes, chromosome abnormalities, mutations affecting protein charge or function, or alteration in sex ratio (Neel *et al.*, 1990) in children of atomic bomb survivors, although the exposure differs from prolonged courses of radiotherapy or chemotherapy.

The sex ratio of offspring might give an indication of genetic damage since lethal mutations on the X chromosome would cause a deficit of sons (Rucknagel, 1981), but neither our data nor the literature (Table III) suggest this occurs.

The excess of twins in our data has not been remarked upon before, but is seen also in the aggregated literature for adult but not childhood cancer patients (Table III). Although based on small numbers, there are reasons why this is plausible. Treatment of Hodgkin's disease in adulthood leads to premature ovarian failure and raised pituitary gonadotrophin levels, which may precede menopause (Schilsky *et al.*, 1981). Treatment in childhood, however, although it may cause ovarian failure soon after treatment (i.e. before adult ages), appears rarely to lead to premature menopause after a period of ovarian function when pregnancy could occur (Sy Ortin *et al.*, 1990). Incidence of dizygotic twinning in the general population rises with maternal age (except beyond age 40), which is believed to relate to rising pituitary gonadotrophin levels (Campbell, 1988). Thus the hormonal and ovarian effects of treatment of Hodgkin's disease in adulthood may be equivalent in terms of twinning aetiology to the hormonal and ovarian state of women reaching natural menopause at ages considerably older than these patients (indeed one of the mothers of twins in our study entered premature menopause after bearing her twins).

Genetic damage by therapy need not lead to any of the above outcomes, and when they do occur they are frequently not caused by genetic damage. Direct examination of chromosome constitutions provides a powerful method to examine whether or not a certain type of transgenerational genetic effect is occurring, especially since examination of parental chromosomes can determine the parental origin of an abnormality. We found no chromosome abnormalities attributable to the treated parent. However, chromosome analysis will not detect subtle changes to the DNA, including conventional mutations. In the general population almost 1% of individuals have a chromosome abnormality when similar levels of banding are used (Jacobs *et al.*, 1992). Our findings enable us to rule out at the 95% level of confidence that more than 8% of offspring in the study group overall would have had abnormalities, and larger percentages for subgroups of the data. About half of the patients had received chemotherapy and had therefore had a large mutagenic dose to the gonads. The other half had radiotherapy alone, to the upper body, so that their gonadal dose will have been far lower, from scattered radiation. Larger studies with chromosome data would be desirable.

In conclusion, our results and the literature so far are reassuring with respect to pregnancies conceived after chemotherapy and radiotherapy, although the numbers studied are not large enough to exclude quite substantial and important risks or to give satisfactory analyses for specific chemotherapeutic regimens. Further surveillance is needed, to increase the number of children on which risk assessments are based, to give sufficient cases for analyses in relation to specific therapeutic agents, and to re-test whether treatment gives rise to twinning.

**Acknowledgements**
We thank British Nuclear Fuels Ltd for funding this study; the patients who allowed us to interview them and, in particular, to take blood samples from their children; Mrs Anna Bradshaw for help in organising the study, and Dr P Hoskin for assistance; the physicians who took blood samples for their help; Mr Z Qiao for computing assistance; Professor PG Smith and Dr B De Stavola for advice; Dr R Fisher and Dr FJ Paradinas for information on the 69 XXY foetus; and Ms A MacFarlane and OPCS for unpublished data on national births by gestation. The Epidemiological Monitoring Unit is funded by the Medical Research Council.

### References

AISNER J, WIERNIK PH AND PEARL P. (1993). Pregnancy outcome in patients treated for Hodgkin's disease. *J. Clin. Oncol.*, **11**, 507-512.

ANDRIEU JM AND OCHOA-MOLINA ME. (1983). Menstrual cycle, pregnancies and offspring before and after MOPP therapy for Hodgkin's disease. *Cancer*, **52**, 435-438.

BAKER JW, MORGAN RL, PECKHAM MJ AND SMITHERS DW. (1972). Preservation of ovarian function in patients requiring radiotherapy for para-aortic and pelvic Hodgkin's disease. *Lancet*, **1**, 1307-1308.

BLATT J, MULVIHILL JJ, ZIEGLER JL, YOUNG RC AND POPLACK DG. (1980). Pregnancy outcome following cancer chemotherapy. *Am. J. Med.*, **69**, 828-832.

BRESLOW NE AND DAY NE. (1987). *Statistical Methods in Cancer Research. Volume II. The Design and Analysis of Cohort Studies.* IARC Scientific Publication No. 82, IARC: Lyon.

CAMPBELL DM. (1988). Aetiology of twinning. In *Twinning and Twins*, MacGillivray I, Campbell DM, Thompson B. (eds) pp. 27-36, John Wiley: Chichester.

CUZICK J. (1985). A Wilcoxon-type test for trend. *Stat. Med.*, **4**, 87-90.

DODDS L, MARRETT LD, TOMKINS DJ, GREEN B AND SHERMAN G. (1993). Case-control study of congenital anomalies in children of cancer patients. *Br. Med. J.*, **307**, 164-168.

DOLL R, EVANS HJ AND DARBY SC. (1994). Paternal exposure not to blame. *Nature*, **367**, 678-680.

DRAPER GJ. (1989). General overview of studies of multigeneration carcinogenesis in man, particularly in relation to exposure to chemicals. In *Perinatal and Multigeneration Carcinogenesis*, Napalkov NP, Rice JM, Tomatis L, Yamasaki H. (eds) pp. 275-288, IARC Scientific Publications No. 96. IARC: Lyon.

GABRIEL DA, BERNARD SA, LAMBERT J AND CROOM III RD. (1986). Oophoropexy and the management of Hodgkin's disease. A reevaluation of the risks and benefits. *Arch. Surg.*, **121**, 1083-1085.

GREEN DM, AND HALL B. (1988). Pregnancy outcome following treatment during childhood or adolescence for Hodgkin's disease. *Ped. Hematol. Oncol.*, **5**, 269-277.

GREEN DM ZEVON MA, LOWRIE G, SEIGELSTEIN N AND HALL B. (1991). Congenital anomalies in children of patients who received chemotherapy for cancer in childhood and adolescence. *N. Engl J. Med.*, **325**, 141-146.

HAWKINS MM. (1991). Is there evidence of a therapy-related increase in germ cell mutation among childhood cancer survivors? *J. Natl Cancer Inst.*, **83**, 1643-1650.

HAWKINS MM AND SMITH RA. (1989). Pregnancy outcomes in childhood cancer survivors: probable effects of abdominal irradiation. *Int. J. Cancer*, **43**, 399-402.

HAWKINS MM, DRAPER GJ AND SMITH RA. (1989). Cancer among 1,348 offspring of survivors of childhood cancer. *Int. J. Cancer*, **43**, 975-978.

HOLMES GE AND HOLMES FF. (1978). Pregnancy outcome of patients treated for Hodgkin's disease. A controlled study. *Cancer*, **41**, 1317-1322.

HORNING SJ, HOPPE RT, KAPLAN HS AND ROSENBERG SA. (1981). Female reproductive potential after treatment for Hodgkin's disease. *N. Engl J. Med.*, **304**, 1377-1382.

JACOBS PA, BROWNE C, GREGSON N, JOYCE C AND WHITE H. (1992). Estimates of the frequency of chromosome abnormalities detectable in unselected newborns using moderate levels of banding. *J. Med. Genet.*, **29**, 103-108.

JANOV AJ, ANDERSON J, CELLA DF, ZUCKERMAN E, KORNBLITH AB, HOLLAND JC, KANTOR AF AND LI FP. (1992). Pregnancy outcome in survivors of advanced Hodgkin disease. *Cancer*, **70**, 688-692.

KINSELLA TJ, TRIVETTE G, ROWLAND J, SORACE R, MILLER R, FRAASS B, STEINBERG SM, GLATSTEIN E AND SHERINS RJ. (1989). Long-term follow-up of testicular function following radiation therapy for early-stage Hodgkin's disease. *J. Clin. Oncol.*, **7**, 718-724.

KNOX EG AND LANCASHIRE RJ. (1991). *Epidemiology of Congenital Malformations.* HMSO: London.

KREUSER ED, XIROS N, HETZEL WD AND HEIMPEL H. (1987). Reproductive and endocrine gonadal capacity in patients treated with COPP chemotherapy for Hodgkin's disease. *J. Cancer Res. Clin. Oncol.*, **113**, 260-266.

LACHER MJ AND TONER K. (1986). Pregnancies and menstrual function before and after combined radiation (RT) and chemotherapy (TVPP) for Hodgkin's disease. *Cancer Invest.*, **4**, 93-100.

LE FLOCH O, DONALDSON SS AND KAPLAN HS. (1976). Pregnancy following oophoropexy and total nodal irradiation in women with Hodgkin's disease. *Cancer*, **38**, 2263-2268.

LI FP, FINE W, JAFFE N, HOLMES GE AND HOLMES FF. (1979). Offspring of patients treated for cancer in childhood. *J. Natl Cancer Inst.*, **62**, 1193-1197.

LI FP, GIMBRERE K, GELBER RD, SALLAN SE, FLAMANT F, GREEN DM, HEYN RM AND MEADOWS AT. (1987). Outcome of pregnancy in survivors of Wilms' tumor. *J. Am. Med. Assoc.*, **257**, 216-219.

LITTLE J AND THOMPSON B. (1988). Descriptive epidemiology. In *Twinning and Twins*. MacGillivray I, Campbell DM, Thompson B (eds), pp. 37-66. John Wiley: Chichester.

MCKEEN EA, MULVIHILL JJ, ROSNER F AND ZARRABI MH. (1979). Pregnancy outcome in Hodgkin's disease. *Lancet*, **2**, 590.

MULVIHILL JJ, McKEEN EA, ROSNER F AND ZARRABI MH. (1987*a*). Pregnancy outcome in cancer patients. Experience in a large cooperative group. *Cancer*, **60**, 1143-1150.

MULVIHILL JJ, MYERS MH, CONNELLY RR, BYRNE J, AUSTIN DF, BRAGG K, COOK JW, HASSINGER DD, HOLMES FF, HOLMES GF, CRAUSS MR, LATOURETTE HB, MEIGS JW, NAUGHTON MD, STEINHORN SC, STRONG LC, TETAMJ AND WEYER PJ. (1987*b*). Cancer in offspring of long-term survivors of childhood and adolescent cancer. *Lancet*, **2**, 813-817.

NEEL JV, SCHULL WJ, AWA AA, SATOH C, KATO H, OTAKE M AND YOSHIMOTO Y. (1990). The children of parents exposed to atomic bombs: estimates of the genetic doubling dose of radiation for humans. *Am. J. Hum. Genet.*, **46**, 1053-1072.

NOMURA T. (1982). Parental exposure to X-rays and chemicals induces heritable tumours and anomalies in mice. *Nature*, **296**, 575-577.

NOMURA T. (1988). X-ray and chemically induced germ-line mutation causing phenotypical anomalies in mice. *Mutat. Res.*, **198**, 309-320.

OFFICE OF POPULATION CENSUSES AND SURVEYS. (1977-94). *Birth Statistics*. Series FMI. HMSO: London.

OFFICE OF POPULATION CENSUSES AND SURVEYS. (1980-86). *Birthweight Statistics*. Monitor series DH3. OPCS: London.

OFFICE OF POPULATION CENSUSES AND SURVEYS. (1988-93). *Mortality Statistics, Perinatal and Infant: Social and Biological Factors, England and Wales 1987-91*. Series DH3 Nos. 21-25. HMSO: London.

OTAKE M, SCHULL WJ AND NEEL JV. (1990). Congenital malformations, stillbirths, and early mortality among the children of atomic bomb survivors: a reanalysis. *Radiat. Res.*, **122**, 1-11.

RUCKNAGEL DL. (1981). Reproductive potential after treatment for Hodgkin's disease. *N. Engl. J. Med.*, **305**, 890-891.

SCHILSKY RL, SHERINS RJ, HUBBARD SM, WESLEY MN, YOUNG RC AND DEVITA VT. (1981). Long-term follow-up of ovarian function in women treated with MOPP chemotherapy for Hodgkin's disease. *Am. J. Med.*, **71**, 552-556.

SENTURIA YD, PECKHAM CS AND PECKHAM MJ. (1985). Children fathered by men treated for testicular cancer. *Lancet*, **2**, 766-769.

SHERMAN SL, PETERSEN MB, FREEMAN SB, HERSEY J, PETTAY D, TAFT L, FRANTZEN M, MIKKELSEN M AND HASSOLD TJ. (1994). Non-disjunction of chromosome 21 in maternal meiosis 1: evidence for a maternal age-dependent mechanism involving reduced recombination. *Hum. Mol. Genet.*, **3**, 1529-1535.

SPECHT L, HANSEN MM AND GEISLER C. (1984). Ovarian function in young women in long-term remission after treatment for Hodgkin's disease stage I or II. *Scand. J. Haematol.*, **32**, 265-270.

SY ORTIN TT, SHOSTAK CA AND DONALDSON SS. (1990). Gonadal status and reproductive function following treatment for Hodgkin's disease in childhood: the Stanford experience. *Int. J. Radiat. Oncol. Biol. Phys.*, **19**, 873-880.

TRASLER JM, HALES BF AND ROBAIRE B. (1985). Paternal cyclophosphamide treatment of rats causes fetal loss and malformations without affecting male fertility. *Nature*, **316**, 144-146.

WEBBER LM AND CARSON M. (1983). Fluorodeoxyuridine synchronisation of bone marrow cultures. *Cancer Genet. Cytogenet.*, **8**, 123-132.

WHITEHEAD E, SHALET SM, BLACKLEDGE G, TODD I, CROWTHER D AND BEARDWELL CG. (1983). The effect of combination chemotherapy on ovarian function in women treated for Hodgkin's disease. *Cancer*, **52**, 988-993.

YOSHIMOTO Y, NEEL JV, SCHULL WJ, KATO H, SODA M, ETO R AND MABUCH K. (1990). Malignant tumors during the first 2 decades of life in the offspring of atomic bomb survivors. *Am. J. Hum. Genet.*, **46**, 1041-1052.